# Influencing factors in the stagnation of the Baiu front that induced heavy rainfall in July 2020 over Kyushu, Japan

**DOI:** 10.1038/s41598-023-41730-0

**Published:** 2023-09-04

**Authors:** Qoosaku Moteki

**Affiliations:** Japan Agency for Marine-Earth Science and Technology (JAMSTEC), 2-15 Natsushima-Cho, Yokosuka City, Kanagawa 237-0061 Japan

**Keywords:** Atmospheric science, Ocean sciences

## Abstract

The influencing factors of the long-term stagnation of the Baiu front that induced heavy rainfall in July 2020 over Kyushu, Japan, were examined. In July 2020, the position of the Baiu front from a weather chart at 130° E remained stationary over Kyushu for 20 days, and that was the longest from 2002 to 2021. By examining a certain index of the Yellow Sea High (YSH), which is a necessary condition for Baiu front stagnation near Kyushu, it was confirmed that in 2020, a positive high-pressure anomaly persisted over the Yellow Sea until the end of July. The YSH maintenance was due to the significant negative sea surface temperature (SST) anomaly over the Yellow Sea. The significant negative SST anomalies in the Yellow Sea were due to vertical mixing in ocean mixed layer in response to the strong winds of the extratropical cyclones. A rapid SST decrease ranging from 0.5 to 1.5 °C/day was repeatedly occurred by ocean mixed layer deepening in association with the passage of the extratropical cyclones. The anomalous long-term stagnation of YSH due to the negative SST anomalies could have caused the Baiu front to remain stagnant over Kyushu until the end of July.

## Introduction

Heavy rainfall events, collectively referred to as the July 2020 heavy rainfall event^1^, occurred over wide areas of western Japan from 3 to 31 July 2020. In association with this heavy rainfall event, the largest 1/3/6/12/24/48/72-h total rainfall amount in observation history was recorded at 8/11/12/14/21/25/31 of 162 stations throughout Kyushu. This series of heavy rainfall events over Kyushu was due to several quasistationary convective bands (QSCBs^2–4^). QSCBs is defined as a band-shaped heavy rainfall area with a length of 50–300 km and a width of 20–50 km. Their stagnation and repeated formation at the same place often caused localized heavy rainfall events inducing severe disasters such as landslides and floods. Araki et al.^4^ investigated the characteristics of atmospheric environmental fields during the presence of QSCBs in the July 2020 heavy rainfall event and compared the characteristics to those during several heavy rainfall events over Kyushu in 2017 and 2018. The number of QSCBs and the occurrence rate of short-term heavy rainfall events due to QSCBs during the 2020 event were significantly higher than those during the 2017 and 2018 events.

Horinouchi et al.^5^ suggested that one of the large-scale environmental factors influencing the July 2020 heavy rainfall event was the intensification of the Silk Road wave train^6^ in Central Asia (50° E–70° E), which resulted in downstream enhancement in the trough over the East China Sea (120° E–130° E). In addition, the 7-day mean precipitation and moisture flux convergence over Kyushu were the greatest over the last 40 and 30 years, respectively. From the perspective of heavy rainfall forecasting, Terasaki and Miyoshi^7^ demonstrated that with the use of 54-km horizontal resolution and 1024 member ensemble forecasts, the probability of heavy rainfall in Kyushu could be captured approximately 5 days before the extreme rainfall disaster in Kumamoto Prefecture occurred on 4 July.

Moreover, air-sea interaction processes played a significant role in the precipitation peak during the latter half of July in 2020. Yamamoto^8^ simulated heavy rainfall to the north of Kyushu on 26 July and noted that the location of heavy rainfall was sensitive to the large SST anomaly in the vicinity of Kyushu. In addition, Baba^9^ indicated that accurate estimation of the latent heat flux using an air–sea coupled model is useful for improving the predictability of Baiu frontal heavy rainfall for extended range forecasts. Due to the long-term stagnation of the Baiu front under these large-scale environmental conditions, the continuous development of many QSCBs may have induced the July 2020 heavy rainfall event. The July 2020 heavy rainfall event was prolonged by a very long stagnation of the Baiu front near Japan. In a press release of the Japan Meteorological Agency (JMA), the following was expressed: “We cannot remember the Baiu front stagnating for such a long time”^10^.

The remaining question addressed here is, what is the fundamental reason for the long-term stagnation of the Baiu front within the same latitudinal zone (30–35° N)? Moteki and Manda^11^ determined that the development of the cold Yellow Sea High (YSH) is necessary for the stagnation of the Baiu front around Kyushu. The YSH is a cold stationary anticyclone that occurs near the sea surface between April and July due to the low sea surface temperature (SST) over the Yellow Sea, which is strongly cooled during winter^11,12^. The YSH is less clear in weather charts than other well-known high-pressure systems (e.g., the Okhotsk High and Pacific High) and is difficult to objectively detect. The SSTs over the Yellow Sea from April to July are significantly lower than the land surface temperatures of the Chinese continent and the Korean Peninsula at the same latitude, and positive pressure anomalies tend to appear over the Yellow Sea. The center of the closed isobars at 4-hPa intervals in the standard JMA weather chart corresponding to the YSH is not always geographically fixed and is zonally shifted at times. Moteki and Manda^11^ defined the YSH index, which combines the sea level pressure (SLP) anomaly and SST threshold, and they further obtained a clear relationship with the meridional migration of the Baiu front. In normal years, the Baiu season around Kyushu ends in the middle of July, and Baiu withdrawal can be explained by the northward migration of the Baiu front due to the YSH decay associated with increasing SSTs over the Yellow Sea.

To explain why the Baiu front continued to stagnate near Kyushu until late July in 2020, it is necessary to clarify the unusual factors that led to YSH maintenance in association with air–sea interactions. The purpose of this study was to clarify the factor impacting Baiu front stagnation, resulting in the July 2020 heavy rainfall event, by focusing on the duration of the variation in the cold stationary anticyclonic YSH and SST over the Yellow Sea.

## Results

### Stagnation of the Baiu front and the Yellow Sea High

**Figure 1:**
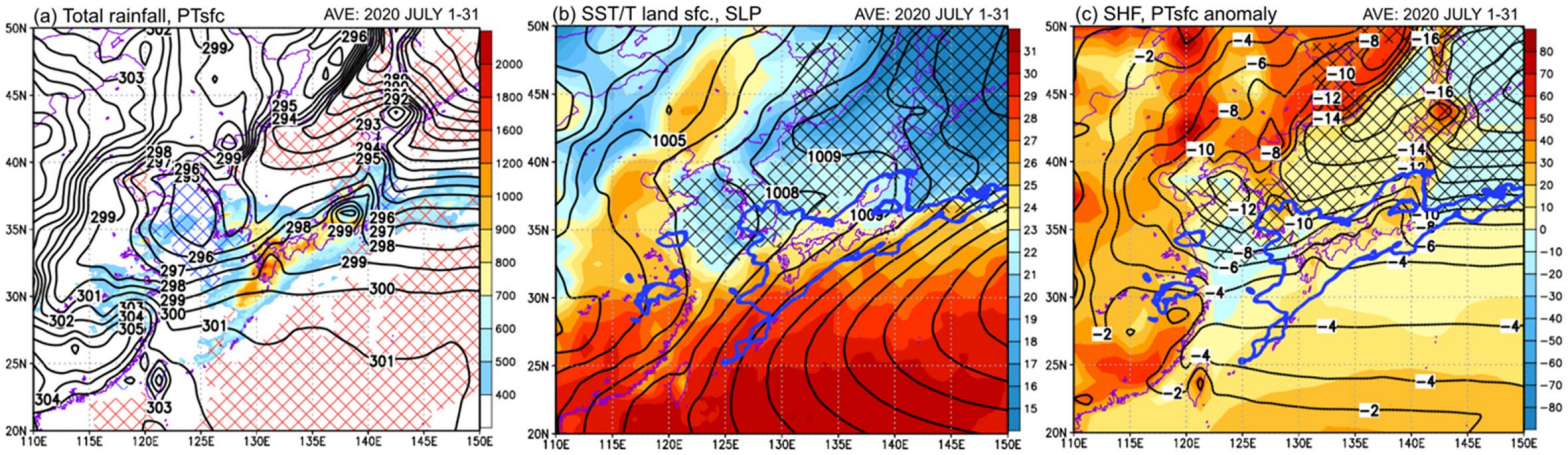
Horizontal distributions of (**a**) total rainfall in July 2020 (mm; shown by the color gradient) and surface potential temperature (K; black contour), (**b**) SST and surface ground temperature (°C; shown by the color gradient) and SLP (hPa; black contour), and (**c**) sensible heat flux (W/m^2^; shown by the color gradient) and surface potential temperature anomaly (K; blue contour) averaged for 1–31 July 2020. The red/blue hatched areas in (**a**) indicate positive/negative SST anomalies exceeding 1 °C. The black hatched areas and blue contours in (**b**) and (**c**) indicate the positive YSH index and total rainfall in July 2020 (500 mm), respectively.

Figure 1 shows the horizontal distributions of the GSMaP monthly accumulated rainfall, SST, and sensible heat flux averaged for July 2020. Large rainfall areas exceeding 500 mm were widely distributed in western Japan at 30–35° N in association with the stagnation of the Baiu front (Fig. 1a). The Baiu frontal heavy rainfall areas corresponded to a strong meridional gradient of the surface potential temperature (296–299 K). In particular, the surface potential temperature was very low over the Yellow Sea (indicated by the closed contours at 295 K), and corresponded to negative SST anomalies below − 1 °C (indicated by the blue hatches). On the other hand, positive SST anomalies above 1 °C (indicated by red hatches) were widely distributed to the south of the Baiu frontal rainfall areas over the Pacific.

In Fig. 1b, the SSTs over the southern part of the Yellow Sea (30–35° N, 120–130° E) showed a strong meridional SST gradient of 21–25 °C, which corresponded well with the strong meridional surface potential temperature gradient shown by the contours in Fig. 1a. The SLP ridge extended east–west at 35–45° N, and the positive YSH indices (indicated by black hatches, see the “Method” section for the definition of the YSH index) were distributed to the north of the Baiu frontal rainfall areas.

The colder anticyclone relative to Chinese continent indicated by the positive YSH index could be due to the zonal contrast of surface heating. As shown in Fig. 1c, compared at the same latitude of 30–40° N, the sensible heat of − 10 to 10 W/m^2^ over the oceanic region was clearly smaller than that of 20–50 W/m^2^ over the Chinese continent, indicating a very large zonal contrast of surface heating. The surface potential temperature anomalies from the zonal mean between 105 and 115° E showed a negative anomaly of − 12 K over the Yellow Sea, indicating that a relatively cold anticyclone of YSH was maintained to the northwest of the heavy rainfall areas in Kyushu.

**Figure 2:**
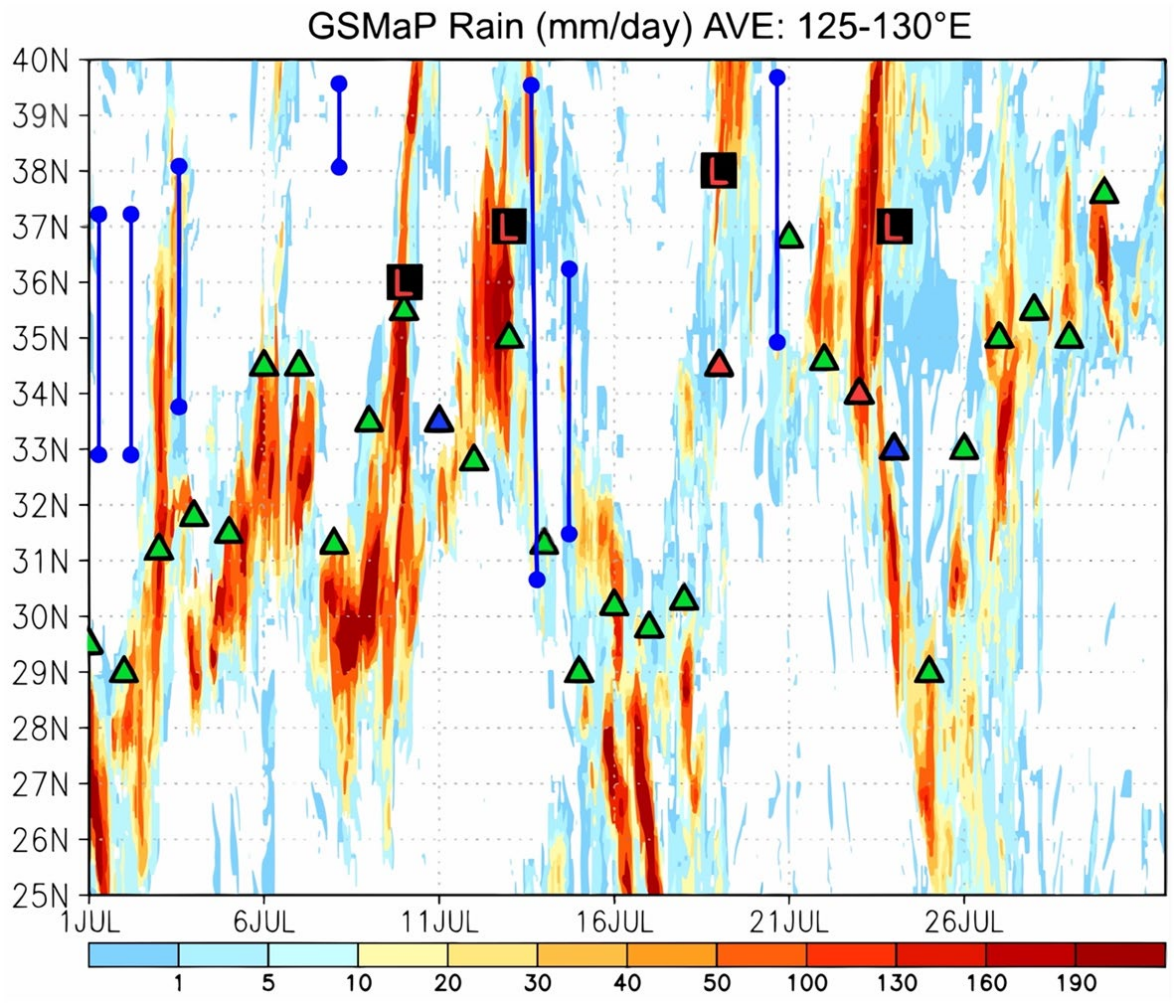
Time–latitude cross section of the GSMaP rainfall intensity (mm/day; shown by the color gradient) for 125–130° E over the period of 1–31 July 2020. The green, red, and blue triangles indicate the meridional positions of the stationary, warm, and cold fronts, respectively, retrieved from the JMA weather chart at 130° E. The red L symbol indicates the passage of an extratropical cyclone over the Yellow Sea (122–125° E), and the blue solid lines indicate the presence of the YSH according to data extracted from the JMA weather chart.

Figure 2 shows the time–latitude cross section of the GSMaP rainfall intensity averaged between 125 and 130° E and the meridional positions of the Baiu front retrieved from the JMA weather chart at 130° E for July 2020. The period of July 1–31 in Fig. 2 is shown to illustrate the fact that precipitation systems associated with the July 2020 heavy rainfall event were continuously present at the same latitude zone (30–35° N). The precipitation systems that produced the heavy rainfall event, originating over the ocean west of Kyushu, are depicted by the rainfall intensity averaged between 125 and 130° E. The date of Baiu withdrawal in 2020 (southern Kyushu: 28 July; northern Kyushu: 30 July) was approximately 2 weeks later than the climatological withdrawal date in the middle of July. Very high rainfall intensities exceeding 100 mm/day (indicated by the red color) continued over Kyushu (30–35° N). Remarkably, the YSH, which contributes to Baiu front stagnation, appeared 7 times (on 1, 2, 4, 8, 14, 15, and 21 July 2020) in the JMA weather chart. Tracking the meridional positions of the Baiu front (the symbols of synoptic fronts during the period between the Baiu onset and withdrawal, which are defined by the JMA) at 130° E showed that the total period during which the Baiu front remained between 30 and 35° N was 20 days (3–9, 11–14, 16, 18–19, 22–24, 26–27, and 29 July), and there was no typhoon around Kyushu in July 2020. The 20 days of Baiu front stagnation in July 2020 was the longest over the 20-year period from 2002 to 2021 (Table 1). Notably, the July 2020 heavy rainfall event that occurred over western Japan from 3 to 31 July was found to be associated with the long-term stagnation of the Baiu front.

**Table 1:** Number of total days with the Baiu front (the symbols of synoptic fronts during the period between the Baiu onset and withdrawal, which are defined by the JMA) between 30 and 35° N at 130° E in July from 2002 to 2021.

yy	'02	'03	'04	'05	'06	'07	'08	'09	'10	'11	'12	'13	'14	'15	'16	'17	'18	'19	'20	'21	Ave	Std
Day	5	19	2	11	17	16	3	11	13	3	6	2	9	7	9	3	5	10	20	5	8.8	5.7

The rightmost columns provide the averages and standard deviations over 20 years.

**Figure 3:**
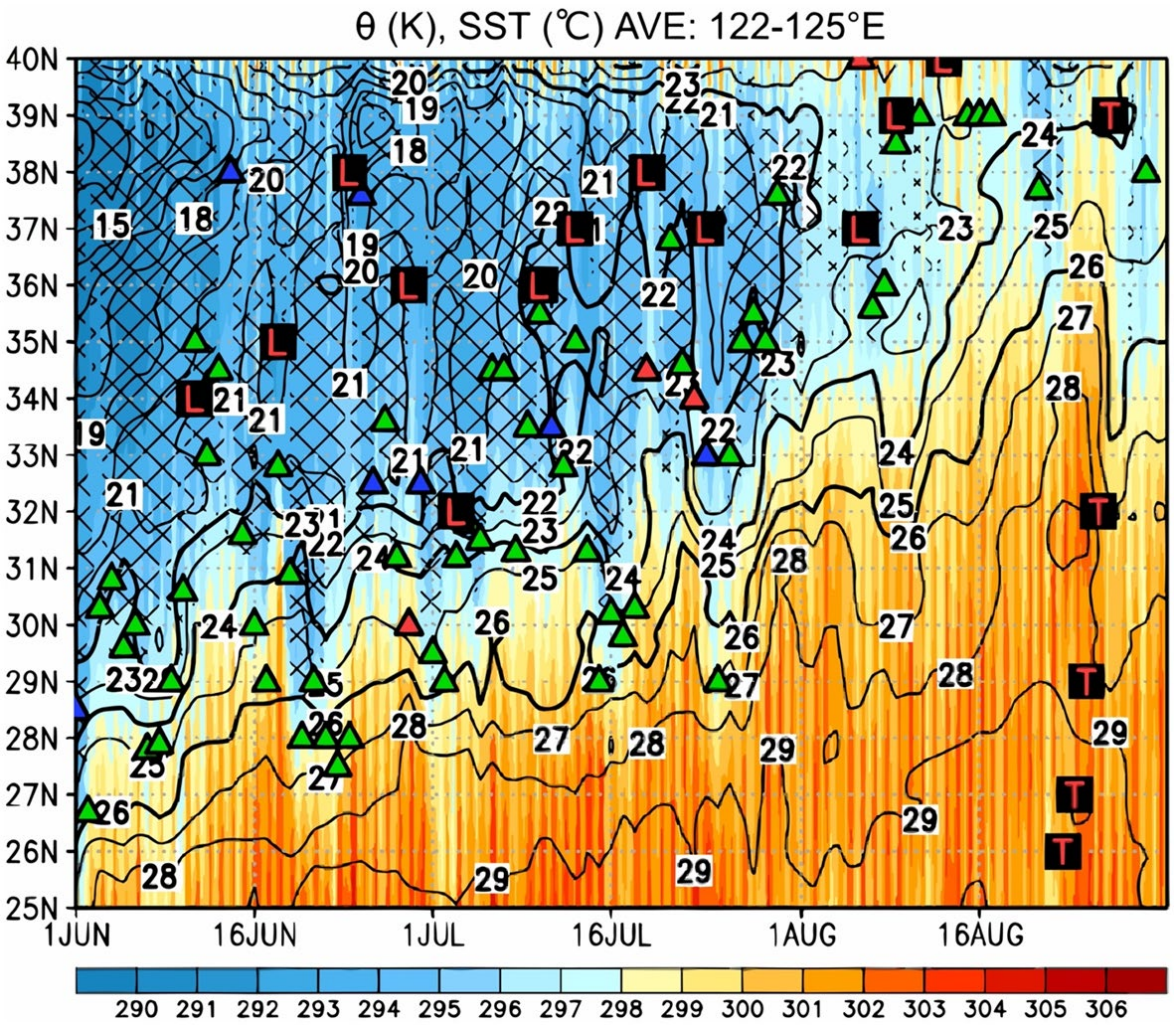
Time–latitude cross section of the surface potential temperature derived from JRA-55 data (K; shown by the color gradient), SST retrieved from the NOAA OIv2.1 product (°C; contour), and positive YSH index (hatched) averaged between 122 and 125° E for the period of 1 June–31 August 2020. The green, red, and blue triangles indicate the meridional positions of the stationary, warm, and cold fronts, respectively, retrieved from the JMA weather chart at 130° E. The red L and T symbols indicate the passage of an extratropical cyclone and typhoon, respectively, over the Yellow Sea (122–125° E).

**Figure 4:**
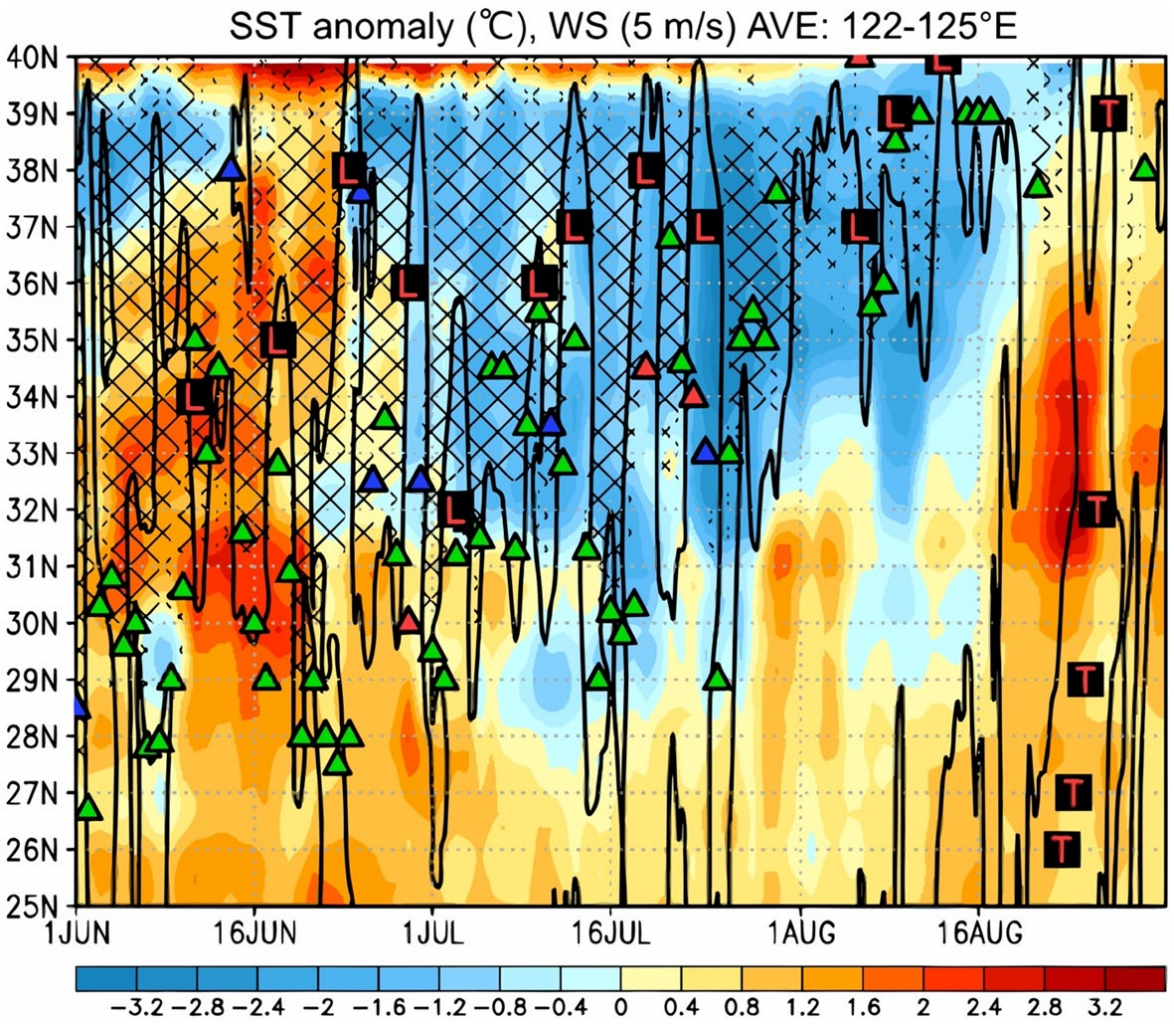
Time–latitude section of the SST anomaly retrieved from the NOAA OIv2.1 product (°C; shown by the color gradient), and positive YSH index (hatched) averaged between 122 and 125° E for the period of 1 June–31 August in 2020. The bold black contour indicates the surface wind speed at 5 m/s obtained from JRA-55. The green, red, and blue triangles indicate the meridional positions of the stationary, warm, and cold fronts, respectively, retrieved from the JMA weather chart at 130° E.

**Figure 5:**
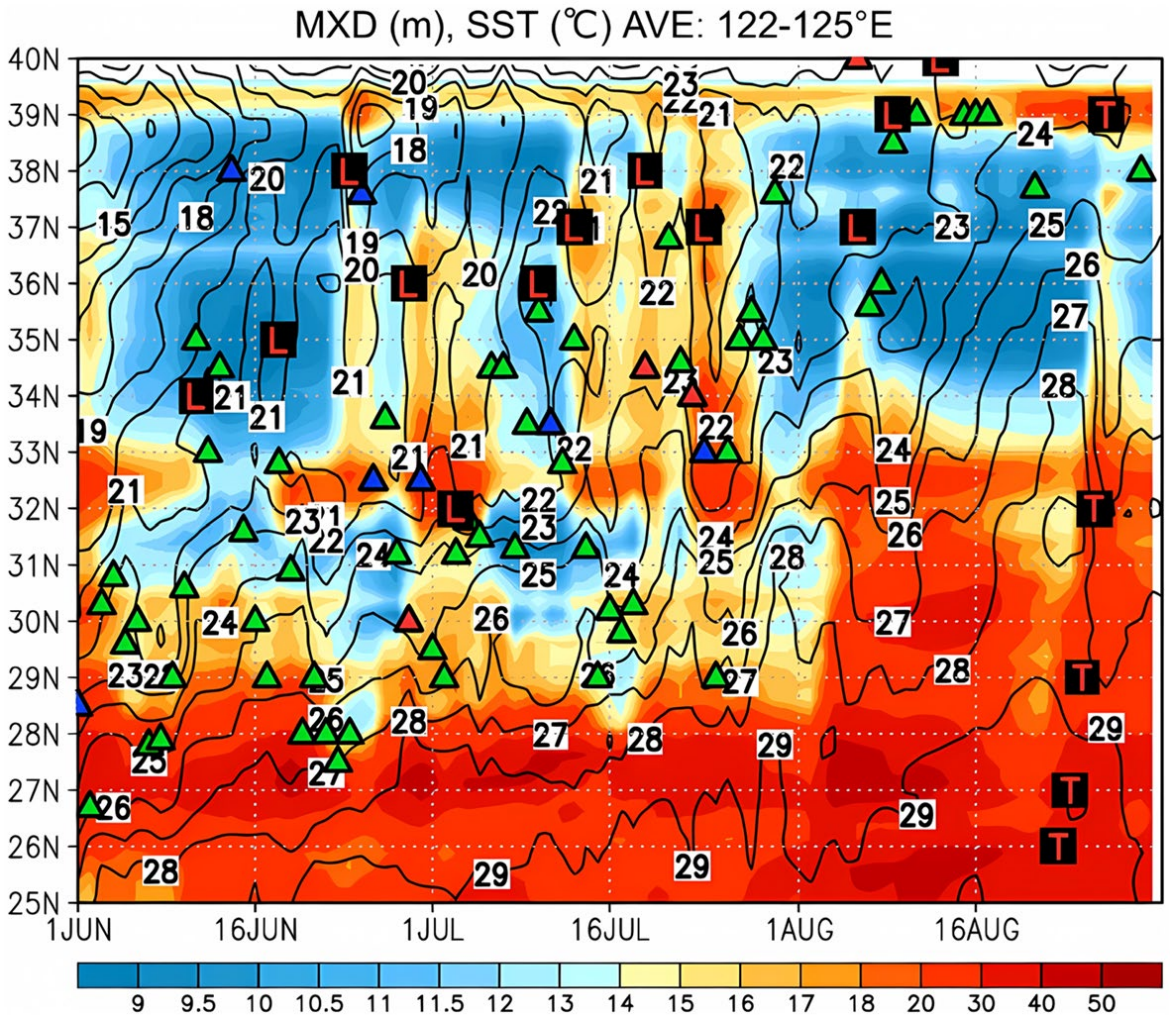
Time–latitude section of the ocean mixed layer depth from ECCO2 data (m; shown by the color gradient) and SST (contour) averaged between 122 and 125° E for the period of 1 June–31 August in 2020. The green, red, and blue triangles indicate the meridional positions of the stationary, warm, and cold fronts, respectively, retrieved from the JMA weather chart at 130° E.

**Figure 6:**
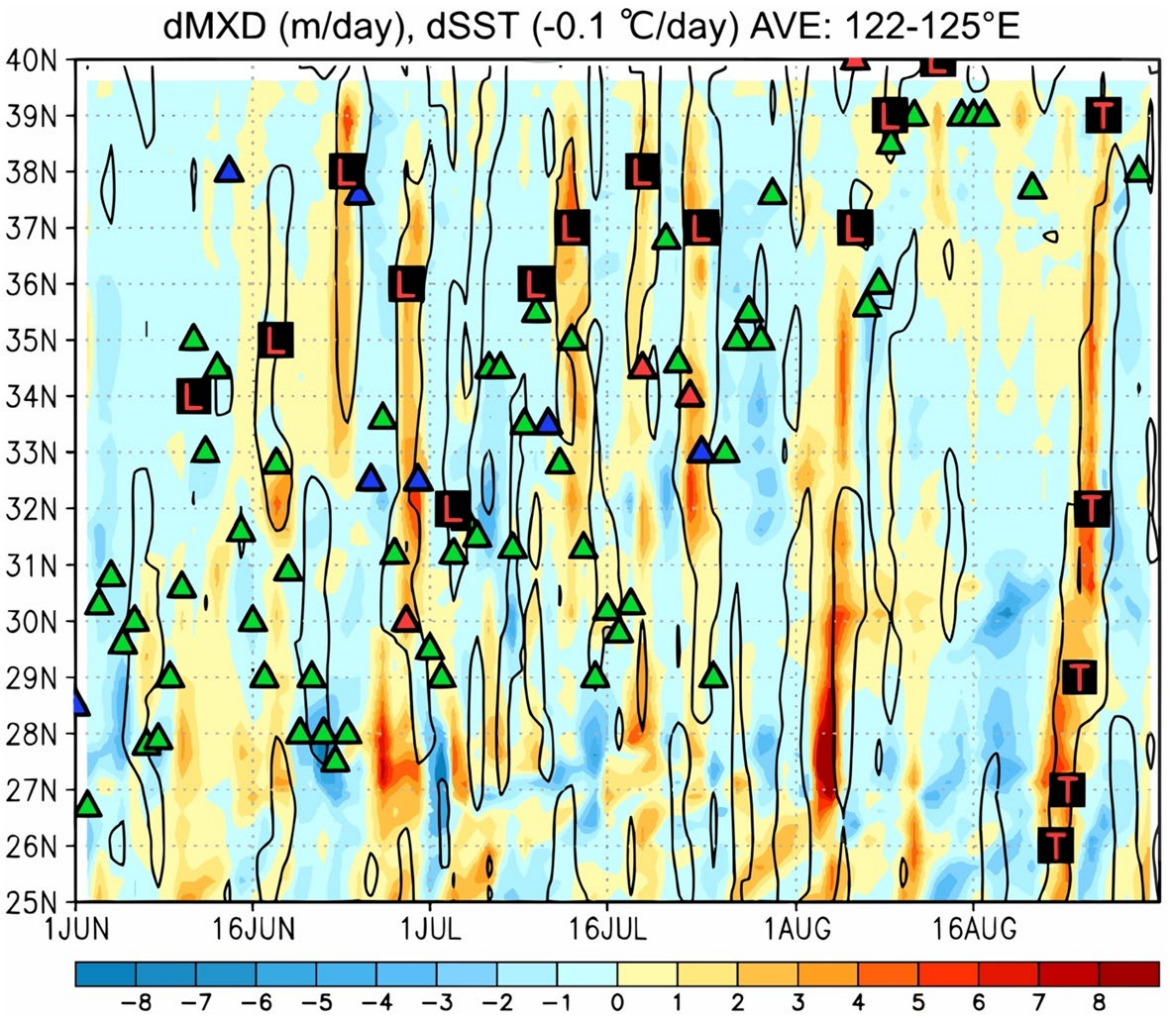
Time–latitude section of the differential ocean mixed layer depth from ECCO2 data (m/day; shown by the color gradient) and 0.1 °C/day decrease in the SST (contour) averaged between 122 and 125° E for the period of 1 June–31 August in 2020. The green, red, and blue triangles indicate the meridional positions of the stationary, warm, and cold fronts, respectively, retrieved from the JMA weather chart at 130° E.

Figure 3 shows the time–latitude cross section of the surface potential temperature, SST, and YSH index for June, July, and August 2020 averaged between 122 and 125° E corresponding to the Yellow Sea and western part of the East China Sea. The period from 1 June to 31 August in Figs. 3, 4, 5, and 6 is shown to illustrate the atmospheric and oceanographic conditions changed before and after the July 2020 heavy rainfall event. During June 2020, the positive SLP anomaly of the YSH index shown in Fig. 3 is observed to the north of 28° N, and the Baiu front is located within the latitudinal zone between 25 and 35° N, as is typical in normal years. During July in normal years, the Baiu front migrates northward and disappears in the middle of July due to the YSH decay. However, during July 2020, the Baiu front remains for longer than that in the normal years, and the positive SLP anomaly of the YSH index continues within the latitudinal zone between 30 and 38° N throughout July 2020. The SST contour representing 24 °C varied between 30 and 33° N, and a notable surface potential temperature contrast was maintained between 30 and 35° N throughout July 2020. The meridional positions of the synoptic front based on the JMA weather chart corresponded to the notable surface potential temperature contrast at the southern edge of the positive YSH index.

Figure 4 shows the same information as Fig. 3 but for the SST anomaly based on the 30-year climatological mean (1981–2010) of NOAA OIv2.1 data and the surface wind speed of 5 m/s retrieved from the JRA-55 data. The minimum and average SST anomalies over the Yellow Sea were approximately − 4 °C and − 1.5 °C, respectively. Generally, the SSTs over the southern Yellow Sea increase during the latter half of July, thereby eventually exceeding 24 °C, and the YSH occurrence frequency significantly decreases^11^. However, in 2020, the SSTs over the whole Yellow Sea remained quite low, i.e., below 24 °C, until the end of July. The extended presence of the YSH, which remained for longer than that in typical years, could be due to these significant negative SST anomalies. The long-term stagnation of the Baiu front is thought to be attributable to YSH maintenance associated with negative SST anomalies. Since the SST over the Yellow Sea, which should normally increase in July, remained quite low (less than 24 °C), the YSH was maintained for a long time, and the Baiu front could not migrate north of 35° N.

Moreover, the SSTs over the Yellow Sea in June were found to be dominated by positive anomalies ranging from 0.4 to 2 °C. After the strong surface southerly winds (indicated by the black contours of 5 m/s in Fig. 4) associated with the passage of a strong extratropical cyclone emerged on 29 June, the positive SST anomalies were drastically reversed into negative anomalies between 32 and 37° N. During the June–August period in 2020, 12 extratropical cyclones and Typhoon Bavi passed over the Yellow Sea (11, 18, 24, and 29 June; 3, 10, 13, 19, and 24 July; and 6, 9, 13, and 23–27 August), and the SSTs were suggested to decrease in association with the strong surface winds.

### Influence of extratropical cyclones on the SST over the Yellow Sea

The SSTs were suggested to decrease in association with the strong surface winds of 12 extratropical cyclones and Typhoon Bavi that passed over the Yellow Sea (11, 18, 24, and 29 June; 3, 10, 13, 19, and 24 July; and 6, 9, 13, and 23–27 August). In particular, the influence of the extratropical cyclone on 29 June was suggested as a cause of the remarkable negative SST anomalies observed in July. The SSTs over the Yellow Sea could drastically decrease due to vertical mixing in the ocean surface layer associated with the strong winds of the extratropical cyclone.

Figure 5 shows the same information as Fig. 3 but for the ocean mixed layer depth obtained from the ECCO2 data. During the first half of June, the depth of the ocean mixed layer was very shallow (~ 10 m), and the SST gradually increased under seasonal migration. During the latter half of June, the observed deepening (> 15 m) of the ocean mixed layer corresponded to low SSTs (21–22 °C) as each extratropical cyclone passed over the Yellow Sea. In July, the ocean mixed layer remained deeper than 15 m because of the passage of the above 5 extratropical cyclones, which inhibited the increase in the SST associated with seasonal migration. In August, the ocean mixed layer became shallower (~ 10 m), and the SST gradually increased again under seasonal migration.

Figure 6 shows the same information as Fig. 3 but for the differential ocean mixed layer depth retrieved from the ECCO2 data. As extratropical cyclones passed on 18, 24, and 29 June and 13, 19, and 24 July and Typhoon Bavi passed from 23 to 27 August over the Yellow Sea, the 1–5 m/day ocean mixed layer deepening was found to greatly correspond to the 0.1 °C/day SST decrease. This fact suggests that the water temperature structure in the ocean surface layer of the Yellow Sea was significantly changed by atmospheric forcing associated with the strong surface winds of the extratropical cyclone.

**Figure 7:**
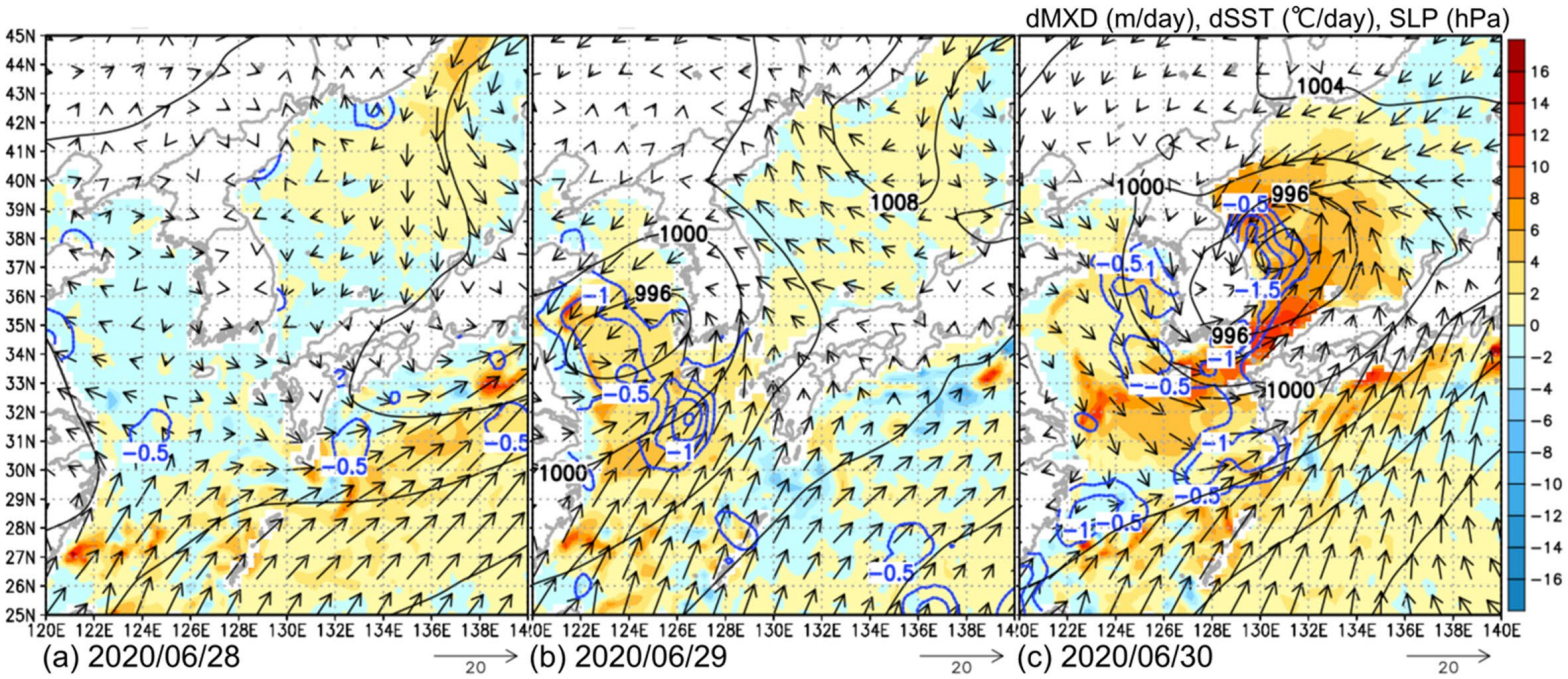
Horizontal distributions of the differential ocean mixed layer depth from ECCO2 data (m/day; shown by the color gradient), daily mean SLP (hPa; black contour), and SST decrease (°C/day; blue bold contour) on (**a**) 28, (**b**) 29, and (**c**) 30 June 2020. The daily averaged winds are shown by the vectors.

Here, in order to examine how the extratropical cyclones affect the ocean surface water temperature structure, the extratropical cyclone passing over the Yellow Sea from 28 to 30 June is selected because it shows the most drastic SST decrease. Figure 7 shows the horizontal distributions of the differential water temperature retrieved from the ECCO2 data and the differential SST for the extratropical cyclone passing over the Yellow Sea from 28 to 30 June. As shown in Fig. 5, the negative SST anomalies in the Yellow Sea during July were likely triggered by the passage of the extratropical cyclone at the end of June. As shown in Fig. 7a 1 day before extratropical cyclone passage, there was almost no variation in both the ocean mixed layer depth and SST under calm conditions. As shown in Fig. 7b, on June 29, when the extratropical cyclone passed through the Yellow Sea, the SST decreased in areas where the ocean mixed layer deepened throughout the entire Yellow Sea. On June 30 (Fig. 7c), the developing extratropical cyclone moved into the Sea of Japan, and the ocean mixed layer deepened under the influence of strong winds within a broader area. The SST significantly decreased in the eastern Yellow Sea, along the north side of the Kuroshio Current, off the east coast of mainland China and southwest of Kyushu. In summary, the ocean mixed layer deepened by 5–15 m, and the SST decreased by 0.5–1.5 °C over the Yellow Sea from June 28 to 30 as this extratropical cyclone passed.

**Figure 8:**
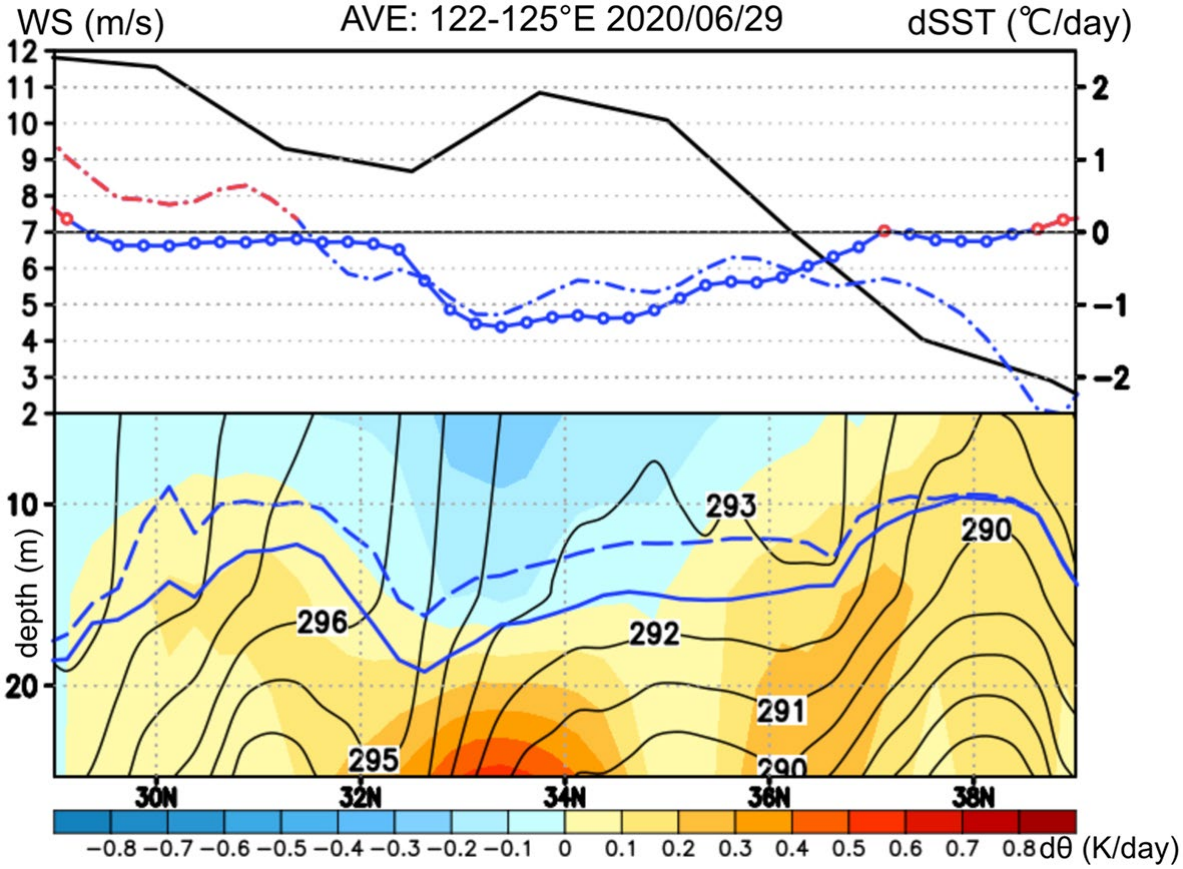
The top Panel (**a**) shows the meridional distribution of the surface wind speed (m/s; black line), SST increase/decrease (°C/day; red/blue line), and positive/negative SST anomaly (°C; red/blue dot-dashed line) averaged between 122 and 125° E on 29 June 2020. The bottom Panel (**b**) shows the vertical section of the differential water potential temperature from ECCO2 data (°C/day; shown by the color gradient) between 27 and 30 June 2020 and water potential temperature (°C; shown by the contours) on 30 June 2020, averaged between 122 and 125° E. The ocean mixed layer depths retrieved from ECCO2 data on 28 and 29 June are shown by the blue dashed and solid lines, respectively.

Figure 8 shows the vertical section of the differential water temperature obtained from the ECCO2 data and the differential SST averaged between 122 and 125° E on 29 June. When the extratropical cyclone passed at 34° N and 124° E, the ocean surface layer of the Yellow Sea indicated that the water temperature structure was significantly impacted in response to the surface wind maximum. As shown in Fig. 8a, the peak of the surface wind speed (9–11 m/s) at approximately 34° N corresponded very well with the observed 1 °C SST decreases and 1 °C negative SST anomalies. As shown in Fig. 8b, the SST decrease was consistent with the decrease in the water temperature within the ocean surface layer (5–15 m depth) and the increase in the thermocline (15–35 m depth). The structure of the water temperature fluctuations suggested that vertical mixing caused the water temperature in the mixed layer to decrease and that in the thermocline to increase as the extratropical cyclone passed. Between 30 and 34° N, where the meridional gradient of the SST was high, vertical mixing could easily occur in response to surface wind forcing. Ekman upwelling associated with horizontal divergence in the ocean surface layer with a 200–300 km scale was locally confirmed in the Yellow Sea and could partially contribute to the observed SST reduction (not shown). However, because the stratification in the ocean surface layer was highly notable in the Yellow Sea, deepening of the ocean mixed layer was the most reasonable process for the broad-area SST decrease.

In contrast, to the south of 30° N, positive SST anomalies were observed despite strong southwesterly winds exceeding 10 m/s. Thus, the sensitivity of the oceanic response to atmospheric forcing was very different between the Yellow Sea and Kuroshio Current because of the difference in the corresponding ocean mixed layer depths. As a result, high SSTs greater than 24 °C were maintained over the Kuroshio Current, while the SST over the Yellow Sea was significantly reduced under extratropical cyclone passage, and the meridional gradient of the SST was enhanced, which was considered to contribute to the prolonged stagnation of the Baiu front.

## Conclusions

In this paper, the factors that contributed to the prolonged stagnation of the Baiu front were examined, inducing heavy rainfall from 3 to 31 July 2020. Tracking the meridional position of the Baiu front from a weather chart at 130° E demonstrated that the front remained stationary between 30 and 35° N for a total of 20 days from 3 to 29 July. The number of total days with Baiu front stagnation in July 2020 was the greatest from 2002 to 2021.

Examining the index of the YSH, which is a necessary condition for Baiu front stagnation in the vicinity of Kyushu, confirmed that the positive high-pressure anomaly over the Yellow Sea in 2020 continued until the end of July. YSH maintenance could have been due to the negative SST anomaly of 1–3 °C occurring over the Yellow Sea throughout July 2020. In addition, the impact of an extratropical cyclone passing at the end of June was suggested as a factor in the occurrence of significant negative SST anomalies in the Yellow Sea. With the passage of the strong extratropical cyclone through the central part of the Yellow Sea from June 29–30 in 2020, a drastic SST decrease of 0.5–1.5 °C/day was caused by ocean mixed layer deepening. The water temperature structure in the ocean surface layer of the Yellow Sea was significantly changed by atmospheric forcing associated with the strong surface winds of the extratropical cyclones. The very strong thermal stratification structure within the ocean surface layer (0–30 m depth) prior to the extratropical cyclone passage was changed by the vertical mixing process associated with atmospheric forcing.

The Kuroshio Current exhibited a deep ocean mixed layer (20–50 m) and achieved a low sensitivity to atmospheric forcing. Therefore, SSTs above 24 °C were maintained, continuously heating and moistening the lower atmosphere. In contrast, the Yellow Sea, with a shallow bathymetry (approximately 50 m depth), exhibited very strong thermal stratification and ocean mixed layer depths below 10 m, resulting in high sensitivity to atmospheric forcing. Thus, the difference in the depth of the oceanic mixing layer between the Kuroshio and the Yellow Sea resulted in distinct sensitivity of the oceanic response to atmospheric forcing (strong surface winds associated with the extratropical cyclones). In addition, 5 extratropical cyclones passed through the Yellow Sea in July 2020, thereby causing repeated ocean mixed layer deepening and the maintenance of negative SST anomalies. The SST over the Yellow Sea remained extremely low (below 22 °C) until the end of July, and the YSH was maintained.

As a result, a notable SST contrast (22–24 °C) was maintained at approximately 30° N because the difference in the SST between the Kuroshio Current and Yellow Sea remained large throughout July 2020. The notable contrasts in the sea surface and air surface temperatures were consistent with YSH maintenance over the sea to the west of Kyushu and could be one of the factors that caused the long-term stagnation of the Baiu front from 30 to 35° N. The pressure trough associated with the stagnation of the Baiu front becomes the storm track for the next extratropical cyclone moving eastward from the China continent. The passages of the extratropical cyclones repeatedly induce different oceanic responses in the Yellow Sea and the Kuroshio, enhancing the meridional SST contrast. Consequently, the Baiu front is likely to remain stagnant for an increasingly longer term.

Previous studies on heavy rainfall during the Baiu season in Kyushu indicated several processes in which the water vapor flux increased due to the high SSTs over the Kuroshio Current and Pacific Ocean^13–18^. However, the results of the present study indicated that it is necessary to consider not only the high SSTs to the south, which contribute to the increase in the water vapor flux but also the low SSTs to the north, which prolong the stagnation of the Baiu front.

Even during the latter half of July, a pressure trough between the Yellow Sea High and the Pacific High could always continue to develop, causing the Baiu front to remain stagnant for a long period. The prolonged stagnation of the Baiu front that induced the heavy rainfall event in July 2020 is found to be caused by different air–sea interaction processes over the Kuroshio Current and the Yellow Sea.

## Methods

### Data

Precipitation estimates were obtained from the Global Satellite Mapping of Precipitation (GSMaP) standard product^19–23^. The spatial resolution of these data were 0.1° × 0.1°, and the temporal resolution was 1 h. Japanese 55-year reanalysis (JRA-55) data, covering the period from 1958 to the present^24,25^, were used to investigate large-scale atmospheric environmental factors. The dataset exhibited a 1.25° horizontal resolution, 38 levels (including the surface and levels from 1 to 1000 hPa), and 6-h intervals. In addition, surface heat flux terms retrieved from land surface analysis in JRA-55 were provided at 3-h intervals. The positions of synoptic-scale fronts were derived from the JMA weather chart, which was archived from 2002 to 2021, at 00 UTC each day with a meridional resolution of 1°. In this study, the Baiu front was defined as all synoptic-scale fronts (including the warm and cold fronts in the JMA weather chart) that emerge during the period between the onset and withdrawal of the Baiu season, as determined by the JMA.

The SST and anomaly from the 30-year climatological mean were obtained from the National Oceanic and Atmospheric Administration (NOAA) Optimal Interpolation (OI) SST product version 2.1 (OIv2.1)^26,27^. A three-dimensional ocean objective analysis of the Estimating the Circulation and Climate of the Ocean-Phase II (ECCO2) was employed to investigate the ocean structure under the YSH. The ECCO2 3-day average dataset contained 50 vertical levels at a maximum model depth of 6150 m with a high global ocean resolution of 0.25° × 0.25°^28^. The ECCO2 provided adjoint method-based state estimates constrained to the available satellite (SST and SSH) and in situ (vertical temperature and salinity profiles) data and is available from 16 September 1992 to the present.

### The definition of the Yellow Sea High

The Yellow Sea High is formed due to the east‒west thermal contrast between the cold SST over the Yellow Sea and the high SAT strongly heated over Chinese continent. Then, Moteki and Manda^11^ define the Yellow Sea High index, which combines the SLP anomaly and SST threshold. The YSH index is defined as a positive SLP anomaly from the zonal mean between 105 and 115° E at the same latitude. The SLP averages for the 105–115° E is the representative SLP over the area strongly heated over the Chinese continent. The east‒west thermal contrast between the Chinese continent and Yellow Sea is detected as the positive SLP anomaly over the cold SST area relative to the SLP averages for the 105–115° E over the continent.

The YSH occurred in areas with a positive SLP anomaly and a surface atmospheric temperature (SAT) below 24 °C. The SAT threshold of 24 °C was because the SST of the Kuroshio Current exceeded 24 °C. The conditions needed for YSH development were lost as the SST over the Yellow Sea increased and exceeded 24 °C, and the SST difference between the Kuroshio Current and Yellow Sea disappeared. The YSH index could be employed to detect the presence of cold anticyclones related not only to remarkable YSH occurrences, as indicated by closed SLP contours but also to cold SLP ridges extending to the east.

## Data Availability

The data supporting the conclusions of this article are available upon request. Please contact the authors for data requests.
